# Association of plasma uric acid with ischaemic heart disease and blood pressure: mendelian randomisation analysis of two large cohorts 

**DOI:** 10.1136/bmj.f4262

**Published:** 2013-07-18

**Authors:** Tom M Palmer, Børge G Nordestgaard, Marianne Benn, Anne Tybjærg-Hansen, George Davey Smith, Debbie A Lawlor, Nicholas J Timpson

**Affiliations:** 1 Division of Health Sciences, Warwick Medical School, University of Warwick, Warwick, UK; 2 Medical Research Council Integrative Epidemiology Unit at the University of Bristol, Bristol BS8 2BN, UK; 3 Department of Clinical Biochemistry and The Copenhagen General Population Study, Herlev Hospital, Copenhagen University Hospital, Copenhagen, Denmark; 4 Faculty of Health and Medical Sciences, University of Copenhagen, Copenhagen, Denmark; 5 The Copenhagen City Heart Study, Frederiksberg Hospital, Copenhagen University Hospital, Denmark; 6 Department of Clinical Biochemistry, Rigshospitalet, Copenhagen University Hospital, Denmark

## Abstract

**Objectives** To assess the associations between both uric acid levels and hyperuricaemia, with ischaemic heart disease and blood pressure, and to explore the potentially confounding role of body mass index.

**Design** Mendelian randomisation analysis, using variation at specific genes (*SLC2A9* (rs7442295) as an instrument for uric acid; and *FTO* (rs9939609), *MC4R* (rs17782313), and *TMEM18* (rs6548238) for body mass index).

**Setting** Two large, prospective cohort studies in Denmark.

**Participants** We measured levels of uric acid and related covariables in 58 072 participants from the Copenhagen General Population Study and 10 602 from the Copenhagen City Heart Study, comprising 4890 and 2282 cases of ischaemic heart disease, respectively.

**Main outcome** Blood pressure and prospectively assessed ischaemic heart disease.

**Results** Estimates confirmed known observational associations between plasma uric acid and hyperuricaemia with risk of ischaemic heart disease and diastolic and systolic blood pressure. However, when using genotypic instruments for uric acid and hyperuricaemia, we saw no evidence for causal associations between uric acid, ischaemic heart disease, and blood pressure. We used genetic instruments to investigate body mass index as a potentially confounding factor in observational associations, and saw a causal effect on uric acid levels. Every four unit increase of body mass index saw a rise in uric acid of 0.03 mmol/L (95% confidence interval 0.02 to 0.04), and an increase in risk of hyperuricaemia of 7.5% (3.9% to 11.1%).

**Conclusion** By contrast with observational findings, there is no strong evidence for causal associations between uric acid and ischaemic heart disease or blood pressure. However, evidence supports a causal effect between body mass index and uric acid level and hyperuricaemia. This finding strongly suggests body mass index as a confounder in observational associations, and suggests a role for elevated body mass index or obesity in the development of uric acid related conditions.

## Introduction

Uric acid is a powerful antioxidant and has been proposed to protect against cardiovascular disease and some cancers.^1^ In humans and great apes, the gene for urease or urate oxidase (which is expressed most in the kidney and liver^2^) is a non-functioning pseudogene. The absence of a functional unit disables this locus and results in uniquely high levels of serum urate, with about 5-25% of humans having impaired renal excretion and ultimately hyperuricaemia.^3^ The relative fitness advantages gained from the antioxidant properties of uric acid have been suggested to explain why the genetic precondition for such levels persists.^4^ ^5^

Despite the expectation that the antioxidant properties of uric acid might have a protective effect against cardiovascular disease, studies have reported associations with a greater risk of ischaemic heart disease, higher blood pressure, and an adverse cardiovascular risk profile.^6^ ^7^ ^8^ ^9^ ^10^ ^11^ ^12^ ^13^ ^14^ These adverse effects have been confirmed in meta-analyses of prospective studies,^13^ ^15^ which concluded that hyperuricaemia was associated with increases in risk for cardiovascular outcomes and blood pressure, independently of established risk factors.

A series of hypotheses has suggested why these unexpected positive associations might exist, including the upregulation of renin release and the subsequent cascade related reduction of endothelial function.^16^ However, in the absence of proven causal associations, a possible explanation could be reverse causality, whereby preclinical atherosclerosis before a diagnosis of ischaemic heart disease could lead to higher levels of uric acid. Confounding could also account for these associations, and it is notable that a pooled adjusted association with ischaemic heart disease was shown to be attenuated by comparison with the unadjusted association. For the incidence of ischaemic heart disease, without adjustment for risk factors of the disease, the relative risk was 1.34 (95% confidence interval 1.19 to 1.49), compared with 1.09 (1.03 to 1.16) after adjustment.^16^

Mendelian randomisation can account for unmeasured confounding and reverse causation by using genotypes robustly associated with the risk factor of interest as instrumental variables. This approach can be used to test and estimate the causal effect between a risk factor and an outcome.^17^ ^18^ The rs7442295 single nucleotide polymorphism in the *SLC2A9* gene (solute carrier family 2, facilitated glucose transporter member 9) has been found to be robustly associated with increased plasma levels of uric acid, hyperuricaemia, urate excretion, and gout in genome wide association studies.^3^ ^19^ As a result, it has been proposed as an instrument for examining the causal effect of uric acid on disease outcomes.^20^ Hypothetically, variation at this locus divides the population into non-confounded groups assigned only by genotype that have differing levels of plasma uric acid and are not subject to reverse causation. Given the properties of these groups, the use of *SLC2A9* variation as a proxy measurement in this way allows for differences in disease risk or measured phenotype across the groups to be attributed solely to the differences in average levels of uric acid attributable to genotypic variation.

We aimed to investigate whether there was a causal effect of uric acid and hyperuricaemia on ischaemic heart disease and diastolic and systolic blood pressure using a mendelian randomisation approach (fig 1). To determine whether body mass index might be a particularly potent confounder of this association, we also examined the effect of body mass index on uric acid levels using genetic variants that have been shown to be robustly associated with body mass index.

**Figure fig1:**
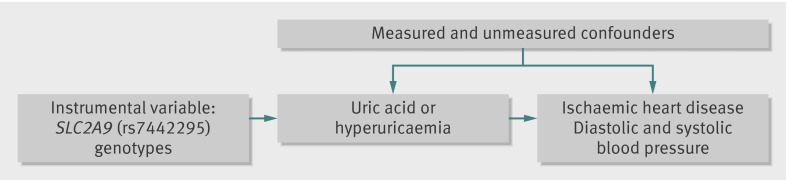
**Fig 1** Directed acyclic graph for instrumental variable analysis using *SLC2A9 *(rs7442295) as an instrument for the effect of uric acid and hypericaemia on ischaemic heart disease and blood pressure

## Methods

### Population

Data were collected from two large cohorts, the Copenhagen General Population Study and the Copenhagen City Heart Study. The studies were approved by the Danish ethical committees and Herlev Hospital, Copenhagen University Hospital, and have been described previously.^21^ All participants in both studies were white and of Danish descent. The Copenhagen General Population Study is a prospective study with participants randomly selected from the Danish Civil Registration System. Eligible participants were aged 20 years and older and resident in greater Copenhagen; ongoing recruitment began in 2003. The Copenhagen City Heart Study is a prospective study of a cohort aged 20 years and older, randomly selected from the population of the city of Copenhagen followed from baseline assessment in 1976-78, with follow-up examinations in 1981-83, 1991-94, and 2001-03. The Copenhagen General Population Study and Copenhagen City Heart Study had 58 072 and 10 602 eligible participants, respectively.

### Ischaemic heart disease

Information on the diagnosis of ischaemic heart disease was collected and verified from 1977 until May 2011 by reviewing all hospital admissions and diagnoses entered in the national Danish Patient Registry and all causes of death entered in the national Danish Causes of Death Registry. Ischaemic heart disease was angina pectoris or myocardial infarction (according to code 410 from ICD-8 (international classification of diseases, eighth revision) and codes I21-I22 from ICD-10), based on characteristic chest pain, electrocardiographic changes, or elevated concentrations of cardiac enzymes after changes in diagnostic criteria over time.^22^ We used ischaemic heart disease rather than myocardial infarction to obtain the greatest possible statistical power. Importantly, however, about 60% of all individuals with ischaemic heart disease also had a myocardial infarction. Follow-up was 100% complete—that is, no individual was lost to follow-up in either study.

### Systolic and diastolic blood pressure

In both studies, systolic and diastolic blood pressure was measured by clinic assessment at baseline, as described previously.^23^ For participants who were receiving antihypertensive treatment, we made adjustments for systolic and diastolic blood pressure by adding a constant value of 10 mm Hg and 5 mm Hg, respectively.^24^

### Body mass index

To exclude the influence of age and sex on our results, we standardised body mass index into age and sex adjusted z scores within each study (web table 1). One z score corresponds to a standard deviation of 4 units of body mass index; thus, for straightforward interpretation of results, estimates in z score units were converted to the scale for body mass index.

### Plasma uric acid

Plasma uric acid was measured by clinic assessment at baseline, using colorimetry on a Konelab Autoanalyser (Thermo Scientific). In the Copenhagen City Heart Study, participants had up to four examinations, and measurements of plasma uric acid were available from the 1991-94 and 2001-03 examinations. Each participant’s measurement of uric acid—and hence hyperuricaemia status—that was recorded closest before their event of ischaemic heart disease was used. Hyperuricaemia was defined as plasma levels of uric acid greater than 0.4 mmol/L (or 6.72 mg/dL, which sits at the approximate limit of solubility for urate).^25^ Plasma uric acid was internally standardised by 5 year age groups and sex using a z score (web table 1) over the whole sample; results were therefore relative risks per one standard deviation increase in uric acid.

### Genotyping

Genotyping was conducted blind to phenotypic data using an ABI PRISM 7900HT sequence detection system (Applied Biosystems) with TaqMan. Genotyping was verified by DNA sequencing in at least 30 individuals, and reruns were performed twice—resulting in successful rates of genotype collection of more than 99.96%. Genotypes of *SLC2A9* (rs7442295) were coded by applying an additive genetic model based on information from a genome wide association study.^19^ We used specific polymorphisms (*FTO *(rs9939609), *MC4R* (rs17782313), and *TMEM18* (rs6548238)) as instruments for body mass index. We selected these polymorphisms because they have the largest known common effect sizes for association with body mass index in European populations.^26^

### Other covariables

In both studies, data were available for age, sex, smoking status, years of education, and income from questionnaires at clinic assessment at baseline. Smoking was categorised as self reported ever smoking. We grouped education according to years spent in school (<10, 10 to <13, and ≥13 years), and annual income by Danish krones (<100 000, 100 000 to 400 000, 400 000 to 600 000, and >600 000). Observational associations were estimated with and without adjustment for potential confounding factors.

### Statistical analysis

For genetic variants, we investigated deviation from the Hardy-Weinberg equilibrium using a Pearson χ^2^ test. Cox proportional hazards regressions was used to estimate observational hazard ratios for ischaemic heart disease per standard deviation change in uric acid concentration, by hyperuricaemia status and by genotype at *SLC2A9 *(rs7442295). We used linear regression to estimate observational associations between blood pressure and uric acid (and therefore hyperuricaemia status), by genotype at *SLC2A9 *(rs7442295). Logistic regression estimated associations between genotype and binary covariates. We used ordered logistic regression to estimate associations between genotype and ordered categorical variables.

Instrumental variable analysis generated causal effects in a mendelian randomisation framework. Estimates of causal hazard ratios for risk of ischaemic heart disease were calculated by using a ratio estimator. These estimates were derived by first dividing the log of the hazard ratio for genotype-ischaemic heart disease by the genotype-exposure coefficient—the resulting value then underwent exponentiation. Standard errors for the log hazard ratios of instrumental variables were derived using the delta method.^27^ Instrumental variable estimation for outcome variables, diastolic blood pressure, systolic blood pressure, uric acid concentration, and hyperuricaemia were also performed using the two stage least squares estimator.^28^

We pooled estimates from the two studies using an inverse variance weighted, meta-analysis model implemented in the user written “metan” Stata command.^29^ Since there were only two studies, we used the fixed effects meta-analysis model. All analyses were performed using Stata (version 11.2).

## Results

The 58 072 eligible participants in the Copenhagen General Population Study included 4890 (8%) with ischaemic heart disease in 141 559 person years at risk, of whom 1682 participants had incident cases. Analyses included 10 602 participants from the Copenhagen City Heart Study from the 1991-94 and 2001-03 examinations, with 2282 (16%) cases of ischaemic heart disease in 79 979 person years at risk, of which 1748 were incident cases.

The mean level of plasma uric acid was 0.30 mmol/L (standard deviation 0.09 mmol/L) in the Copenhagen General Population Study, and 0.31 mmol/L (0.09 mmol/L) in the Copenhagen City Heart Study. Following the definition of hyperuricaemia (uric acid >0.4 mmol/L), 6929 (12%) and 1569 (16%) participants were classified as having hyperuricaemia in the Copenhagen General Population Study and Copenhagen City Heart Study, respectively. Mean diastolic blood pressure was slightly lower in the Copenhagen General Population Study than in the Copenhagen City Heart Study, while mean systolic blood pressure was slightly higher in the Copenhagen General Population Study than in the Copenhagen City Heart Study (table 1).

**Table 1 tbl1:** Baseline characteristics of participants

	Copenhagen General Population Study (n=58 072)	Copenhagen City Heart Study (n=10 602)
Ischaemic heart disease		
Prevalent cases	3208 (6)	534 (5)
Incident cases	1682 (3)	1748 (16)
Total	4890 (8)	2282 (22)
Uric acid (mmol/L)	0.30 (0.09)	0.31 (0.09)
Hyperuricaemia	6929 (12)	1569 (16)
Mean blood pressure (mm Hg)		
Diastolic	84 (12)	86 (12)
Systolic	143 (23)	140 (18)
Body mass index	26 (4)	25 (4)
Age (years)	57 (14)	60 (16)
Male sex	25 921 (45)	4652 (44)
Ever smoked	34 844 (60)	5823 (56)
Education (years)		
<10	17 071 (30)	5393 (52)
10 to <13	30 983 (54)	3856 (37)
≥13	9717 (17)	1195 (11)
Income (Danish krones)		
<100 000	1130 (2)	1884 (18)
100 000 to 400 000	22 518 (39)	5641 (54)
400 000 to 600 000	22 742 (40)	2539 (24)
>600 000	10 785 (19)	334 (3)

Data are mean (standard deviation) for continuous variables and number (%) of non-missing observations for each binary variable.

### Observational estimates of association between uric acid, ischaemic heart disease, and blood pressure

Based on observational estimates, an increase in uric acid of one standard deviation was associated with hazard ratios for ischaemic heart disease of 1.55 (95% confidence interval 1.51 to 1.59) and 1.34 (1.28 to 1.39) in the Copenhagen General Population Study and Copenhagen City Heart Study, respectively. These ratios gave a pooled estimate of 1.49 (1.46 to 1.52; fig 2). This association was attenuated after adjustment for age, sex, smoking, education, and income (pooled hazard ratio 1.21 (1.18 to 1.24)). We found similar patterns for associations of hyperuricaemia with ischaemic heart disease (estimate of pooled hazard ratio for exposed participants 2.21 (2.08 to 2.33); fig 3). This hazard ratio attenuated to 1.41 (1.32 to 1.51) on adjustment for age, sex, smoking, education, and income. In the sensitivity analysis using incident cases of ischaemic heart disease only, estimates of disease risk were attenuated but showed the same pattern across the Copenhagen General Population Study and Copenhagen City Heart Study (web fig 1).

**Figure fig2:**
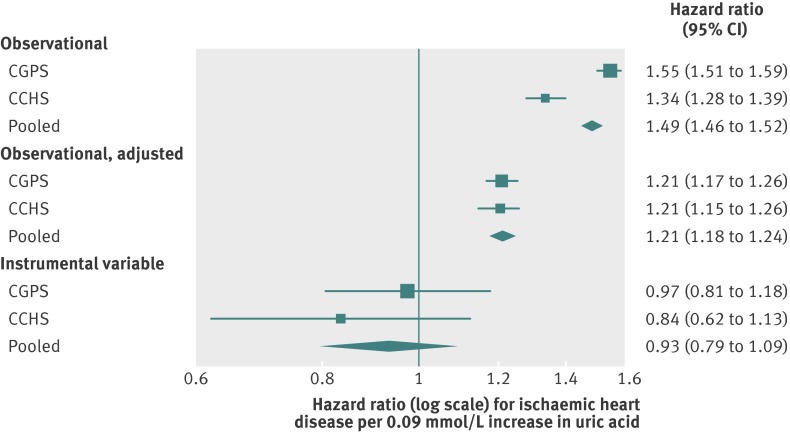
**Fig 2** Forest plot showing observational and instrumental variable estimates of the effect of standardised urate on ischaemic heart disease. Observational adjusted estimates adjusted for age, sex, smoking, education, and income. CGPS=Copenhagen General Population Study; CCHS=Copenhagen City Heart Study; 0.09 mmol/L change in uric acid represents one standard deviation

**Figure fig3:**
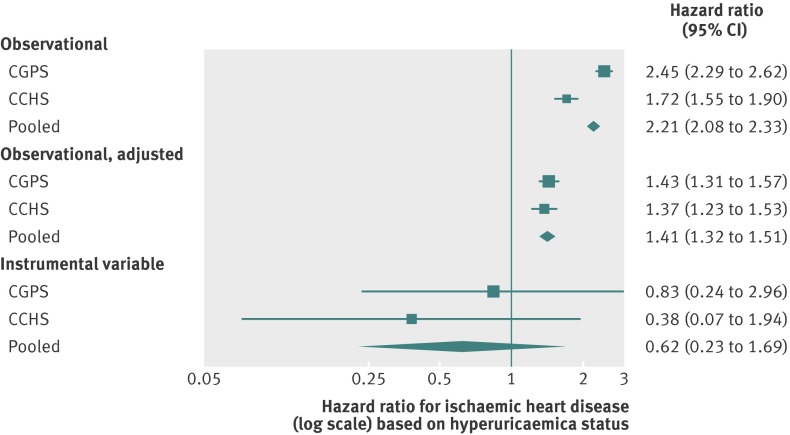
**Fig 3** Forest plot showing observational and instrumental variable estimates of the effect of hyperuricaemia status on ischaemic heart disease. Observational adjusted estimates adjusted for age, sex, smoking, education, and income. CGPS=Copenhagen General Population Study; CCHS=Copenhagen City Heart Study; 0.09 mmol/L change in uric acid represents one standard deviation

A standard deviation increase in uric acid level was associated with an elevation in diastolic blood pressure of 2.42 mm Hg (95% confidence interval 2.32 to 2.51, pooled estimate; web fig 2A). This association was attenuated, after adjustment, to an increase of 1.56 mm Hg (1.45 to 1.67). Participants with hyperuricaemia had an average increase of 4.61 mm Hg (4.31 to 4.90) in diastolic blood pressure (pooled estimate; web fig 2B). This estimate also attenuated on adjustment (2.66 (2.36 to 2.96)). A similar pattern of observational associations was seen between uric acid and hyperuricaemia and systolic blood pressure (web fig 3).

### Association between *SLC2A9* (rs7442295), uric acid and hyperuricaemia, and potential confounding factors

Both the Copenhagen General Population Study and Copenhagen City Heart Study showed no strong evidence for genotypes including *SLC2A9 *(rs7442295) that deviated from the Hardy-Weinberg equilibrium (web table 2). The association between variation at* SLC2A9* (rs7442295) and uric acid was roughly linear (web fig 4). Mean levels of uric acid showed an increase in standard deviation of 0.26 (95% confidence interval 0.24 to 0.27) and 0.25 (0.21 to 0.28) for each additional A allele in the Copenhagen General Population Study and Copenhagen City Heart Study, respectively (table 2). *SLC2A9* (rs7442295) accounted for about 2% of the variance in uric acid levels. Variation at* SLC2A9* (rs7442295) was associated with an increase of about 5% in the risk of hyperuricaemia in both samples.

**Table 2 tbl2:** Associations of *SLC2A9* (rs7442295)* with exposures and outcomes

	Copenhagen General Population Study (n=58 072)				Copenhagen City Heart Study (n=10 602)		
	Hazard ratio (95% CI)	F	R^2^		Hazard ratio (95% CI)	F	R^2^
**Exposures**							
Standardised uric acid	0.26 (0.24 to 0.27)	1304	0.022		0.25 (0.21 to 0.28)	204	0.020
Hyperuricaemia	0.04 (0.03 to 0.04)	272	0.005		0.05 (0.03 to 0.06)	52	0.005
**Outcomes**							
Ischaemic heart disease	0.99 (0.95 to 1.04)	—	—		0.96 (0.89 to 1.03)	—	—
Blood pressure (mm Hg)							
Diastolic	0.11 (−0.07 to 0.29)	1	<0.0001		0.40 (0.00 to 0.80)	4	0.0004
Systolic	0.17 (−0.17 to 0.51)	1	<0.0001		0.12 (−0.50 to 0.73)	0.1	<0.0001
Body mass index	−0.01 (−0.07 to 0.05)	0.1	<0.0001		−0.07 (−0.20 to 0.06)	1	0.0001

Data are hazard ratio (95% confidence interval), F statistic from analysis of variance F test, and R^2^ coefficient of determination.

*Genotypes under an additive model.

In the Copenhagen General Population Study and Copenhagen City Heart Study, *SLC2A9* (rs7442295) was not associated with potential confounders: age, sex, body mass index, smoking, education, and income (web table 2). By contrast, ischaemic heart disease, systolic blood pressure, and diastolic blood pressure showed strong associations with these potential confounders (web table 3). Uric acid and hyperuricaemia exposures also showed associations with these potential confounders (web table 4).

### Association of *SLC2A9* (rs7442295) with health outcomes

Variation at *SLC2A9* (rs7442295) was not associated with ischaemic heart disease in either the Copenhagen General Population Study (hazard ratio 0.99 (95% confidence interval 0.95 to 1.04)) or the Copenhagen City Heart Study (0.96 (0.89 to 1.03)). Similarly, we did not find any associations between *SLC2A9* (rs7442295) and either diastolic or systolic blood pressure (table 2).

### Instrumental variable estimates of the causal effect of uric acid and hyperuricaemia on ischaemic heart disease, diastolic blood pressure, and systolic blood pressure

Instrumental variable analysis, when pooled, gave an estimate of the causal hazard ratio for ischaemic heart disease of 0.93 (95% confidence interval 0.79 to 1.09) per standard deviation increase in uric acid (fig 2). The corresponding estimate of the effect of hyperuricaemia status on risk of ischaemic heart disease was 0.62 (0.23 to 1.69; fig 3). In the sensitivity analysis using incident cases of ischaemic heart disease only, we found weaker hazard ratios per standard deviation increase in uric acid and hyperuricaemia (0.87 (0.69 to 1.09) and 0.42 (0.10 to 1.68), respectively; web fig 1).

Instrumental variable estimates of the causal effect of uric acid on diastolic blood pressure failed to show convincing evidence of an effect and had wide confidence intervals. A standard deviation increase in uric acid was estimated to increase diastolic blood pressure by 0.63 mm Hg (95% confidence interval −0.04 to 1.29). This point estimate was largely driven by the association in Copenhagen City Heart Study, and was weaker than the adjusted observational estimate (1.56 mm Hg (1.45 to 1.67); web fig 2A). Similarly, owing to a stronger effect in the Copenhagen City Heart Study, the instrumental variable estimate showed an increase of 4.56 mm Hg (0.01 to 9.12) in diastolic blood pressure in patients with hyperuricaemia (web fig 2B).

We saw no evidence of a causal effect of uric acid or hyperuricaemia on systolic blood pressure (web fig 3). The instrumental variable estimate of the causal effect of uric acid on systolic blood pressure, when pooled, was 0.65 mm Hg (95% confidence interval −0.54 to 1.85) per standard deviation increase in uric acid. In the Copenhagen General Population Study, the instrumental variable estimate of the causal effect of hyperuricaemia on systolic blood pressure was similar to the observational estimates. However, this instrumental variable estimate was much smaller in the Copenhagen City Heart Study, producing a pooled estimate of 4.31 (−3.72 to 12.33).

### Mendelian randomisation analysis of the causal effect of body mass index on uric acid and hyperuricaemia

Observational estimates showed an association of body mass index, a potential confounding factor, with both standardised uric acid and hyperuricaemia. This association remained on adjustment for potential confounding factors. For example, a standard deviation elevation in uric acid was associated with a unit increase of 1.06 (95% confidence interval 1.03 to 1.09) in body mass index, which increased to 1.43 (1.40 to 1.47) on adjustment for the other potential confounders (web fig 5). Furthermore, when assessing the observational relations between uric acid and hyperuricaemia with risk of ischaemic heart disease, diastolic blood pressure, and systolic blood pressure, inclusion of body mass index as an additional covariable resulted in the attenuation of association. Specifically, associations for a standard deviation elevation in uric acid were reduced to a hazard ratio for ischaemic heart disease of 1.16 (1.12 to 1.19), a change of 0.60 mm Hg (0.49 to 0.71) in diastolic blood pressure, and a change of 1.37 mm Hg (1.18 to 1.56) in systolic blood pressure. Hyperuricaemia was associated with a hazard ratio for ischaemic heart disease of 1.30 (1.21 to 1.39), a change of 1.03 mm Hg (0.74 to 1.33) in diastolic blood pressure, and a change of 2.41 mm Hg (1.91 to 2.91) in systolic blood pressure (pooled estimates) after accounting for body mass index (fig 2 and 3; web figs 2 and 3).

In a mendelian randomisation analysis, we reciprocally^30^ assessed whether uric acid had a causal effect on body mass index and whether body mass index had a causal effect on uric acid. Evidence of a causal association between body mass index and uric acid and hyperuricaemia was only found in the direction implicating body mass index as a causal factor. Instrumental variable estimates of the effect of body mass index on uric acid and hyperuricaemia indicated that a standard deviation increase in body mass index (that is, 4 units) causes an increase of 0.03 mmol/L (95% confidence interval 0.02 to 0.04) in uric acid and a 7.5% increase (3.9% to 11.1%) in risk of hyperuricaemia (figs 4 and 5). By contrast, instrumental variable estimates of the effect of either uric acid or hyperuricaemia status on body mass index, which used genotypes with *SLC2A9 *(rs7442295) as instruments for these measures, did not suggest a causal effect on body mass index (web fig 5).

**Figure fig4:**
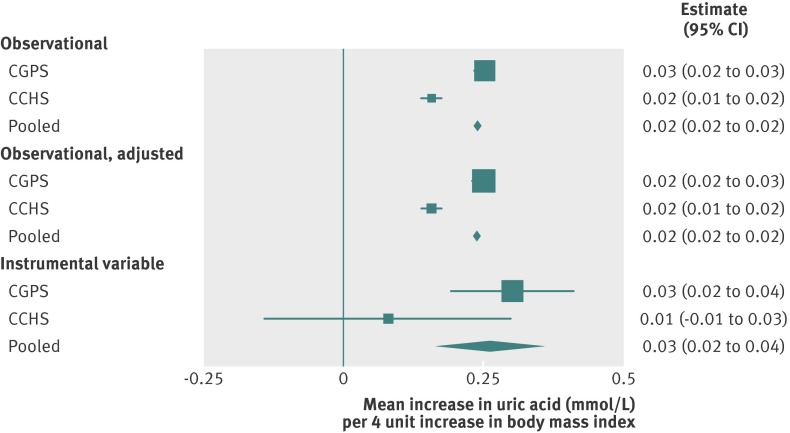
**Fig 4** Forest plot of observational and instrumental variable estimates of a 4 unit increase in body mass index on standardised urate converted to mmol/L. Observational adjusted estimates adjusted for age, sex, smoking, education, and income. CGPS=Copenhagen General Population Study; CCHS=Copenhagen City Heart Study

**Figure fig5:**
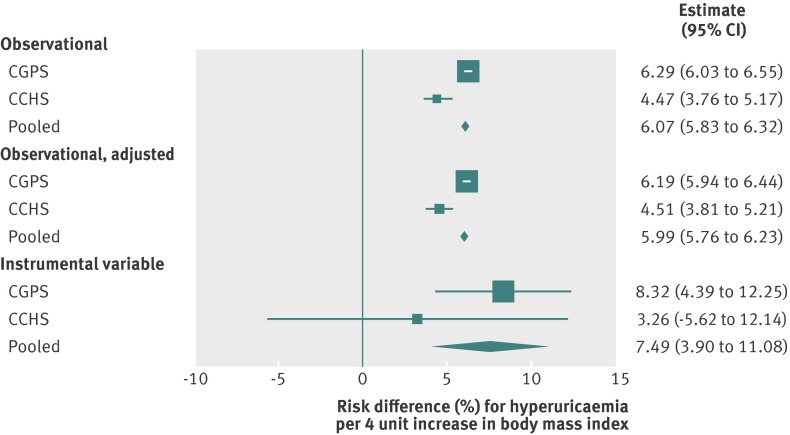
**Fig 5** Forest plot of observational and instrumental variable estimates of a 4 unit increase in body mass index on hyperuricaemia status. Observational adjusted estimates adjusted for age, sex, smoking, education, and income. CGPS=Copenhagen General Population Study; CCHS=Copenhagen City Heart Study

## Discussion

Using data from two large prospective cohort studies, we found no evidence of a causal effect of uric acid or hyperuricaemia on the risk of ischaemic heart disease. Unadjusted observational analyses found an increase of 34-55% in risk of ischaemic heart disease per standard deviation increase in uric acid. These estimates attenuated slightly on adjustment for established cardiovascular risk factors. The same trend was present in our estimates of the association of uric acid and hyperuricaemia on systolic blood pressure. However, mendelian randomisation estimates showed no evidence of an effect of elevated uric acid (and hyperuricaemia) on the risk of ischaemic heart disease or elevated blood pressure.

In view of the weak evidence supporting causal associations, further explanation for known observational associations was sought through the analysis of body mass index as a confounding factor. Body mass index is an established risk factor for both blood pressure and ischaemic heart disease observationally,^31^ ^32^ and there is causal evidence pertaining to the effect of body mass index on ischaemic heart disease, dystolic blood pressure, and systolic blood pressure.^33^ ^34^ It was anticipated and subsequently shown that body mass index exerts a causal and independent effect on levels of uric acid and the risk of hyperuricaemia. Indeed, these instrumental variable estimates fall into the context of a broader literature on the observational association between elevated body mass index and uric acid levels, including that using a mendelian randomisation approach.^35^ ^36^ ^37^

### Comparison with other studies

The mendelian randomisation estimates presented here broadly agree with several recent studies. Stark and colleagues found that *SLC2A9* (rs7442295) was not directly associated with coronary artery disease in a case-control study.^38^ Furthermore, plasma uric acid was not associated with coronary heart disease or ischaemic heart disease in two large cohort studies.^39^ ^40^ More recent evidence using *SLC2A9* variation as an instrument for plasma uric acid failed to find evidence of a causal effect of uric acid on metabolic syndrome,^41^ and further direct analyses have found that *SLC2A9* variants are unlikely to be associated with blood pressure.^42^ Use of a genetic score for hyperuricaemia in a mendelian randomisation approach investigating its association with gout, blood pressure, glucose, chronic kidney disease, and coronary heart disease also revealed no evidence of causal effects.^43^ Despite these findings, another *SLC2A9* locus (rs16890979) has been associated with blood pressure,^44^ suggesting that there is a need to undertake further analyses concerning the possible role of this genomic region and transport system.

### Strengths and limitations of the study

Analyses were undertaken within large, ethnically homogeneous, clinically assessed case series with access to control sets of comparable quality. The nature and size of the existing sample place it particularly well for the undertaking of mendelian randomisation experiments. However, the potential for the complicating effects of linkage disequilibrium, pleiotropy, or the nature of genotypic effect are difficult to account for.^45^ But in favour of the use of *SLC2A9 *variation as an instrument for uric acid levels, this approach does encode for a high capacity sugar transporter, and functional analyses have shown it to have a preferential action on urate.^3^ Yet despite this advantage, causal variants for the reliable effects on uric acid levels have yet to be fully established, and the role of differential isoforms of this gene in the transport of sugars overall could be complex. 

Another potential limitation was that we did not have data available to identify participants receiving uric acid lowering drugs at the time of measurement. Although this will have been a small fraction of our total sample size and unlikely to have materially altered our main results, it may have led to underestimates of both observational and instrumental variable estimates.

### Conclusions and clinical implications of results

Overall, mendelian randomisation estimates found no evidence of causal effects of either uric acid or hyperuricaemia on risk of either ischaemic heart disease or raised blood pressure. Our Mendelian randomisation results alone suggest that uric acid is of limited clinical interest in ischaemic heart disease or blood pressure. However, there is strong evidence for an effect of body mass index on both uric acid and hyperuricaemia—indicating that body mass index is an important confounding factor to observational association analyses of uric acid and hyperuricaemia. This finding contrasts the notion of body mass index operating as a mediator or being on the causal pathway from uric acid to vascular outcomes. In this case, one would expect to find evidence for a causal association both for body mass index and uric acid level, as opposed to just one of these risk factors. 

This study clearly shows the value of mendelian randomisation to help dissect complex networks of risk factor association by using independent proxies or instrumental variables for risk factors of interest. Furthermore, our findings suggest that interventions to reduce body mass index could help improve management of gout and related conditions such as urolithiasis.

**What is already known on this topic**

Uric acid has been suggested to protect against cardiovascular disease
Observational studies have suggested that increased levels of uric acid is associated with ischaemic heart disease and blood pressure; but owing to confounding, bias, and reverse causation, such associations can be difficult to interpret
The *SLC2A9 *gene has been reliably associated with circulating levels of uric acid, and has been proposed as an instrument to investigate causal associations with blood pressure and ischaemic heart disease

**What this study adds**

Genetic variation at the *SLC2A9 *gene shows little evidence of a causal association between increased levels of uric acid, raised blood pressure, and risk of ischaemic heart disease
However, causal analysis of body mass index shows strong evidence of an effect of body mass index on uric acid levels, suggesting considerable confounding in observational associations
Mendelian randomisation analysis suggests that uric acid is of limited clinical interest in ischaemic heart disease or blood pressure. But interventions to reduce body mass index could help improve the management of gout and related conditions such as urolithiasis

## Notes

We thank Dorthe Uldall Andersen for her excellent genotyping, and the staff and participants of the Copenhagen General Population Study and Copenhagen City Heart Study for their important contributions.

## Notes

Cite this as: *BMJ* 2013;347:f4262
