# Supplementary material for: Association of plasma uric acid with ischaemic heart disease and blood pressure: mendelian randomisation analysis of two large cohorts
Source: BMJ. 2013 Jul 18;347:f4262. doi: 10.1136/bmj.f4262 (PMC3715134; doi:10.1136/bmj.f4262)
Supplement: Supplementary file 1 [file palt008481.ww_default.pdf]

Association of plasma uric acid with ischemic heart disease and blood pressure: Mendelian randomization analysis of two large cohorts.

Tom M Palmer, Børge G Nordestgaard, DSc; Marianne Benn, Anne Tybjærg-Hansen, George Davey Smith, Debbie A Lawlor, Nicholas J Timpson.

Supplementary material

Correspondence to Nicholas John Timpson, Medical Research Council Integrative Epidemiology Unit, School of Social and Community Medicine, University of Bristol, Bristol UK, BS8 2BN. Fax:0117 93310123, Tel: 0117 93310131, email:n.j.timpson@bristol.ac.uk.

Supplementary Table 1. Means and standard deviations of body mass index (kg/m^2) by 5 year age band and sex used to generate standardized BMI (Z-scores) in the CGPS and CCHS.

	**CGPS**		**CCHS**	
	**Male**	**Female**	**Male**	**Female**
**5 year age band**	**Mean (SD)**	**Mean (SD)**	**Mean (SD)**	**Mean (SD)**
20-24	24.0 (3.7)	23.7 (4.8)	23.2 (2.7)	22.6 (3.0)
25-29	25.5 (3.9)	24.1 (4.4)	23.6 (2.9)	22.3 (2.9)
30-34	26.0 (3.7)	24.3 (4.3)	24.3 (3.3)	23.0 (3.8)
35-39	26.1 (3.6)	24.5 (4.7)	23.8 (3.4)	22.7 (3.5)
40-44	26.4 (3.6)	25.0 (4.5)	24.2 (3.6)	22.4 (3.5)
45-49	26.8 (3.9)	25.2 (4.6)	24.4 (3.5)	23.1 (4.2)
50-54	26.8 (3.8)	25.4 (4.4)	24.9 (3.7)	23.5 (4.0)
55-59	27.2 (3.9)	26.0 (4.9)	25.3 (3.7)	24.1 (4.5)
60-64	27.4 (3.9)	26.0 (4.6)	25.5 (3.7)	23.9 (4.2)
65-69	27.3 (3.7)	26.3 (4.7)	25.7 (3.4)	24.5 (3.9)
70-74	27.3 (3.7)	26.6 (4.5)	26.0 (3.5)	24.0 (4.0)
75-79	26.9 (3.6)	26.3 (4.4)	26.3 (3.3)	24.7 (3.8)
80-84	26.4 (3.5)	26.1 (4.3)	25.9 (3.0)	24.7 (3.8)
85-99	26.0 (3.3)	25.5 (4.2)	25.3 (2.8)	24.8 (3.8)
Overall	26.9 (3.8)	25.7 (4.6)	25.3 (3.5)	23.9 (4.0)

CGPS: Copenhagen General Population Study; CCHS: Copenhagen City Heart Study; SD: standard deviation.

Supplementary Table 2. Associations of *SLC2A9*(rs7442295) genotype with potential confounders.

	GG	AG	AA	P for trend*	Coefficient/OR (95% CI)†
**CGPS**					
N	2,493 (4%)	19,322 (33%)	36,257 (62%)		
Age, yr	56.7 (56.2, 57.2)	57.1 (56.9, 57.3)	57.1 (57.0, 57.7)	0.25	0.11 (-0.08, 0.30)
Male	1,098 (44%)	8,731 (45%)	16,092 (44%)	0.26	0.98 (0.96, 1.01)
Ever smoked	1,526 (61%)	11,520 (60%)	21,798 (60%)	0.85	1.00 (0.97, 1.03)
Education‡	2	2	2	0.66	0.99 (0.97, 1.02)
Income§	3	3	3	0.32	0.99 (0.96, 1.01)
Body mass index, kg/m^2	26.3 (26.2, 26.5)	26.2 (26.1, 26.3)	26.2 (26.2, 26.3)	0.67	-0.01 (-0.07, 0.05)
**CCHS**					
N, %	454 (4%)	3,534 (33%)	6,614 (62%)		
Age, yr	59.8 (58.3, 61.3)	60.5 (59.9, 61.0)	60.4 (60.0, 60.8)	0.74	0.09 (-0.44, 0.62)
Male	190 (42%)	1,583 (45%)	2,879 (44%)	0.67	0.99 (0.92, 1.05)
Ever smoked	235 (52%)	1,918 (55%)	3,670 (56%)	0.07	1.06 (0.99, 1.14)
Education‡	2	2	2	0.90	1.00 (0.94, 1.07)
Income§	2	2	2	0.04	1.07 (1.00, 1.14)
Body mass index, kg/m^2	24.8 (24.4, 25.1)	24.5 (24.4, 24.7)	24.5 (24.4, 24.6)	0.30	-0.07 (-0.20, 0.06)

Continuous values given as means (95% CI), binary variables given as N (%), categorical variables given as mean category.

Odds ratios for Education and Income from ordered logistic regression.

*In row 'N' P for trend is P for Hardy-Weinberg Equilibrium.

†Assuming an additive model for *SLC2A9* genotype. Estimate is coefficient for age, and odds ratios for male, ever smoked, education, and income.

‡Education coded 1,2,3 indicating <10, 10-13, >13 years of education.

§Income coded 1,2,3,4 indicating <100,000kr, 100,000-400,000kr, 400,000-600,000kr,>600,000kr per year.

CGPS: Copenhagen General Population Study; CCHS: Copenhagen City Heart Study; CI: confidence interval; OR: odds ratio.

Supplementary Table 3. Associations of potential confounders with outcomes.

	CGPS (N_{max}=58,072)	CCHS (N_{max}=10,602)
**Ischemic heart disease**	**HR (95% CI)**	**HR (95% CI)**
Age, yr	2.87 (2.82, 2.92)	1.15 (1.14, 1.17)
Male	1.87 (1.77, 1.98)	1.77 (1.62, 1.92)
Ever smoked	1.61 (1.51, 1.72)	1.42 (1.30, 1.55)
Education	0.65 (0.62, 0.69)	0.69 (0.63, 0.75)
Income	0.62 (0.59, 0.65)	0.88 (0.83, 0.94)
**Diastolic blood pressure (mmHg)**	**Coefficient (95% CI)**	**Coefficient (95% CI)**
Age, yr	0.14 (0.13, 0.15)	0.21 (0.20, 0.23)
Male	3.53 (3.32, 3.74)	4.62 (4.17, 5.08)
Ever smoked	0.12 (-0.10, 0.33)	-0.65 (-1.11, -0.19)
Education	-1.77 (-1.93, 1.61)	-3.32 (-3.65, -2.99)
Income	-0.47 (-0.60, -0.33)	0.47 (0.15, 0.78)
**Systolic blood pressure (mmHg)**	**Coefficient (95% CI)**	**Coefficient (95% CI)**
Age, yr	0.68 (0.66, 0.69)	0.41 (0.39, 0.43)
Male	5.44 (5.05, 5.83)	6.59 (5.89, 7.29)
Ever smoked	1.16 (0.76, 1.56)	-0.92 (-1.63, -0.21)
Education	-7.31 (-7.60, 7.02)	-5.19 (-5.70, -4.69)
Income	-5.22 (-5.47, -4.97)	-0.59 (-1.07, -0.11)
**Body mass index (kg/m^2)**	**Coefficient (95% CI)**	**Coefficient (95% CI)**
Age, yr	0.0002 (-0.002, 0.003)	0.0005 (-0.004, 0.005)
Male	0.00 (-0.066, 0.066)	0.00 (-0.15, 0.15)
Ever smoked	0.08 (0.01, 0.15)	-0.68 (-0.83, -0.53)
Education	-0.61 (-0.65, -0.56)	-0.73 (-0.84, -0.62)
Income	-0.45 (-0.49, -0.41)	-0.12 (-0.22, -0.01)

CGPS: Copenhagen General Population Study; CCHS: Copenhagen City Heart Study; CI: confidence interval; HR: hazard ratio.
Education coded 1,2,3 indicating <10, 10-13, >13 years of education.
Income coded 1,2,3,4 indicating <100,000kr, 100,000-400,000kr, 400,000-600,000kr,>600,000kr per year.

Supplementary Table 4. Associations of potential confounders with exposures (standardized uric acid and hyperuricemia).

	CGPS (N_{max}=58,072)	CCHS (N_{max}=10,602)
**Standardized uric acid**	**Coefficient (95% CI)**	**Coefficient (95% CI)**
Age, yr.	0.01 (0.01, 0.01)	0.009 (0.008, 0.010)
Male	1.02 (1.01, 1.04)	0.84 (0.81, 0.88)
Ever smoked	0.16 (0.15, 0.18)	0.11 (0.07, 0.14)
Education	-0.21 (-0.22, -0.19)	-0.16 (-0.19, -0.13)
Income	-0.08 (-0.09, -0.07)	0.02 (-0.00, 0.05)
Body mass index, kg/m^2	0.070 (0.068, 0.072)	0.043 (0.038, 0.048)
**Hyperuricemia**	**OR (95% CI)**	**OR (95% CI)**
Age, yr.	1.03 (1.03, 1.03)	1.02 (1.01, 1.02)
Male	6.56 (6.16, 6.98)	4.68 (4.14, 5.29)
Ever smoked	1.48 (1.40, 1.56)	1.12 (1.00, 1.25)
Education	0.67 (0.65, 0.70)	0.77 (0.71, 0.83)
Income	0.80 (0.77, 0.82)	1.08 (1.00, 1.16)
Body mass index, kg/m^2	1.14 (1.13, 1.14)	1.08 (1.07, 1.09)

CGPS: Copenhagen General Population Study; CCHS: Copenhagen City Heart Study; CI: confidence interval; OR: odds ratio.
Education coded 1,2,3 indicating <10, 10-13, >13 years of education.
Income coded 1,2,3,4 indicating <100,000kr, 100,000-400,000kr, 400,000-600,000kr,>600,000kr per year.

Supplementary Figure 1. Forest plots of observational and instrumental variable estimates of A) the effect of standardized urate and B) the effect of hyperuricemia status on ischemic heart disease using incident cases only.

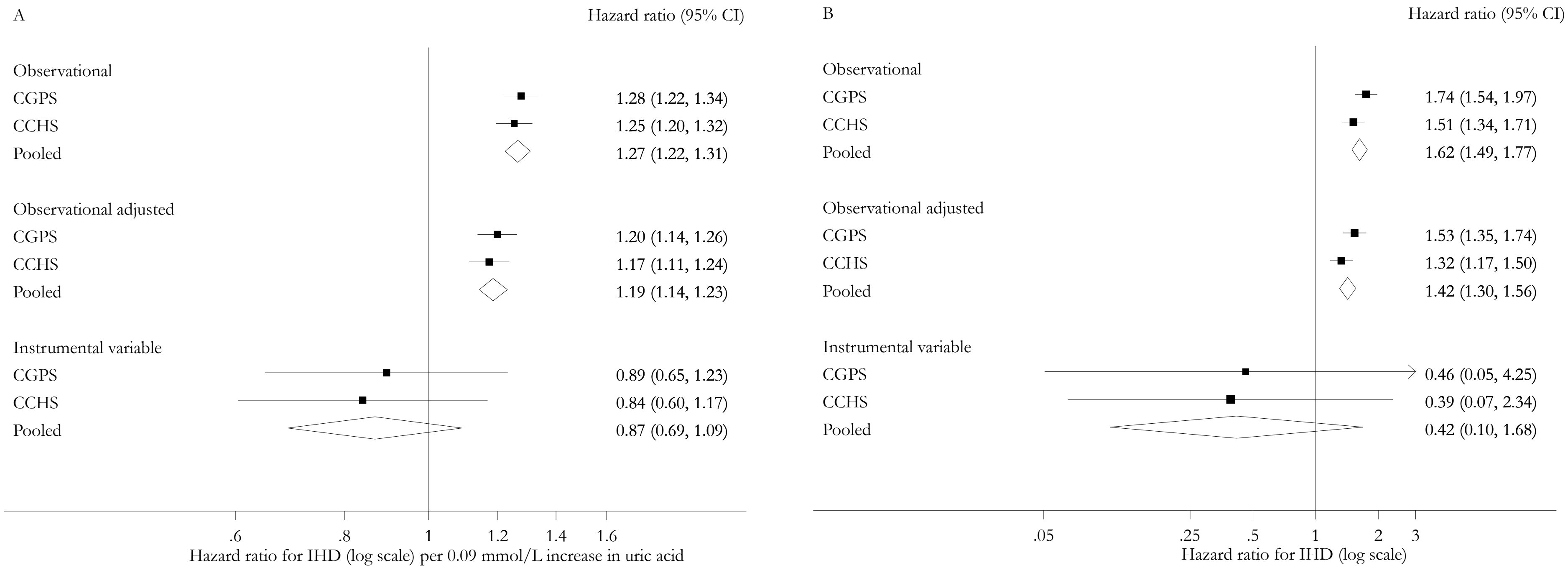

Observational adjusted estimates adjusted for age, sex, smoking, education, and income. CGPS: Copenhagen General Population Study; CCHS: Copenhagen City Heart Study; CI: confidence interval.

Supplementary Figure 2. Forest plots of observational and instrumental variable estimates of A) the effect of standardized urate and B) the effect of hyperuricemia status on diastolic blood pressure (mmHg).

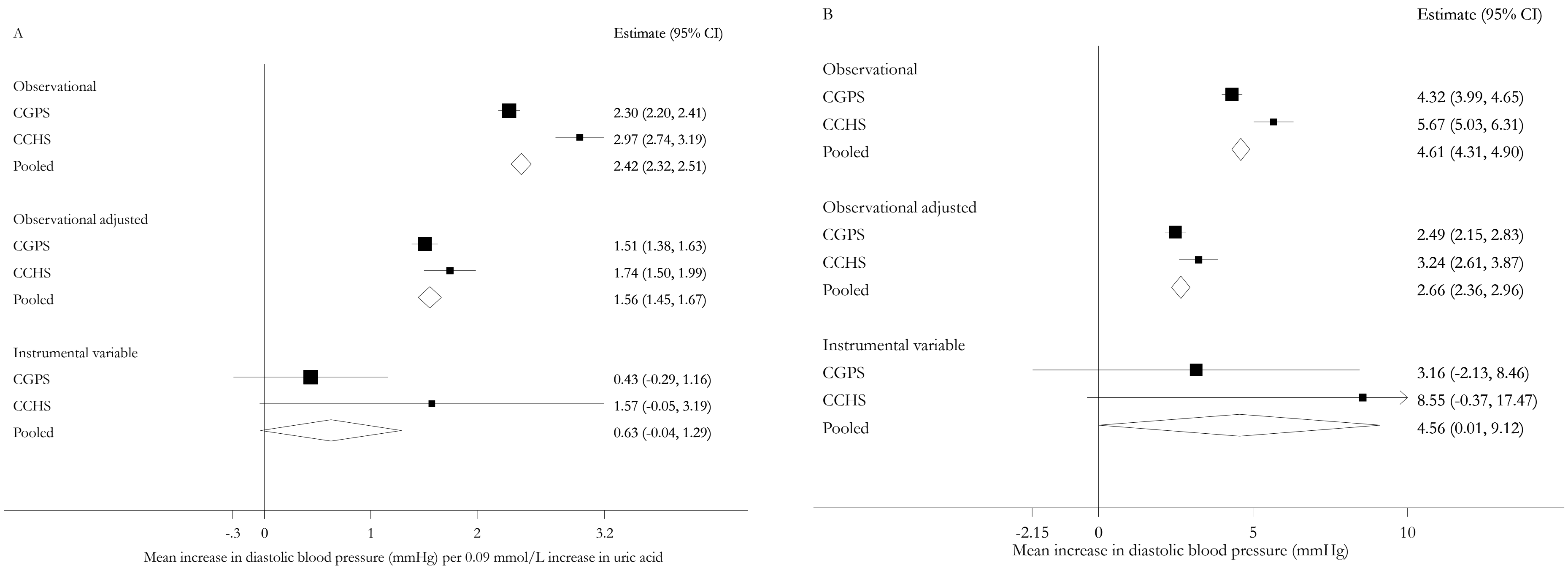

Observational adjusted estimates adjusted for age, sex, smoking, education, and income. CGPS: Copenhagen General Population Study; CCHS: Copenhagen City Heart Study; CI: confidence interval.

Supplementary Figure 3. Forest plots of observational and instrumental variable estimates of A) the effect of standardized urate and B) the effect of hyperuricemia status on systolic blood pressure (mmHg).

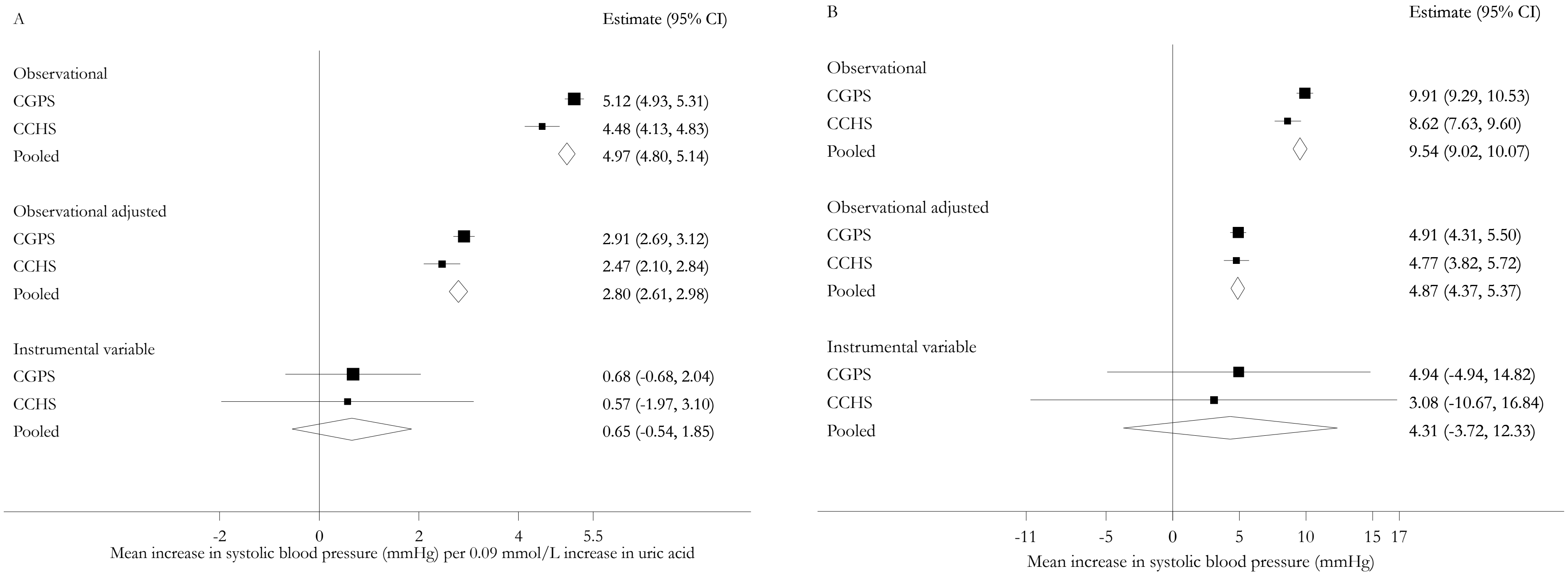

Observational adjusted estimates adjusted for age, sex, smoking, education, and income. CGPS: Copenhagen General Population Study; CCHS: Copenhagen City Heart Study; CI: confidence interval.

Supplementary Figure 4. Mean uric acid by *SLC2A9*(rs7442295) genotype in the Copenhagen General Population Study (CGPS) and Copenhagen City Heart Study (CCHS). Means (points), 95% confidence intervals (bars).

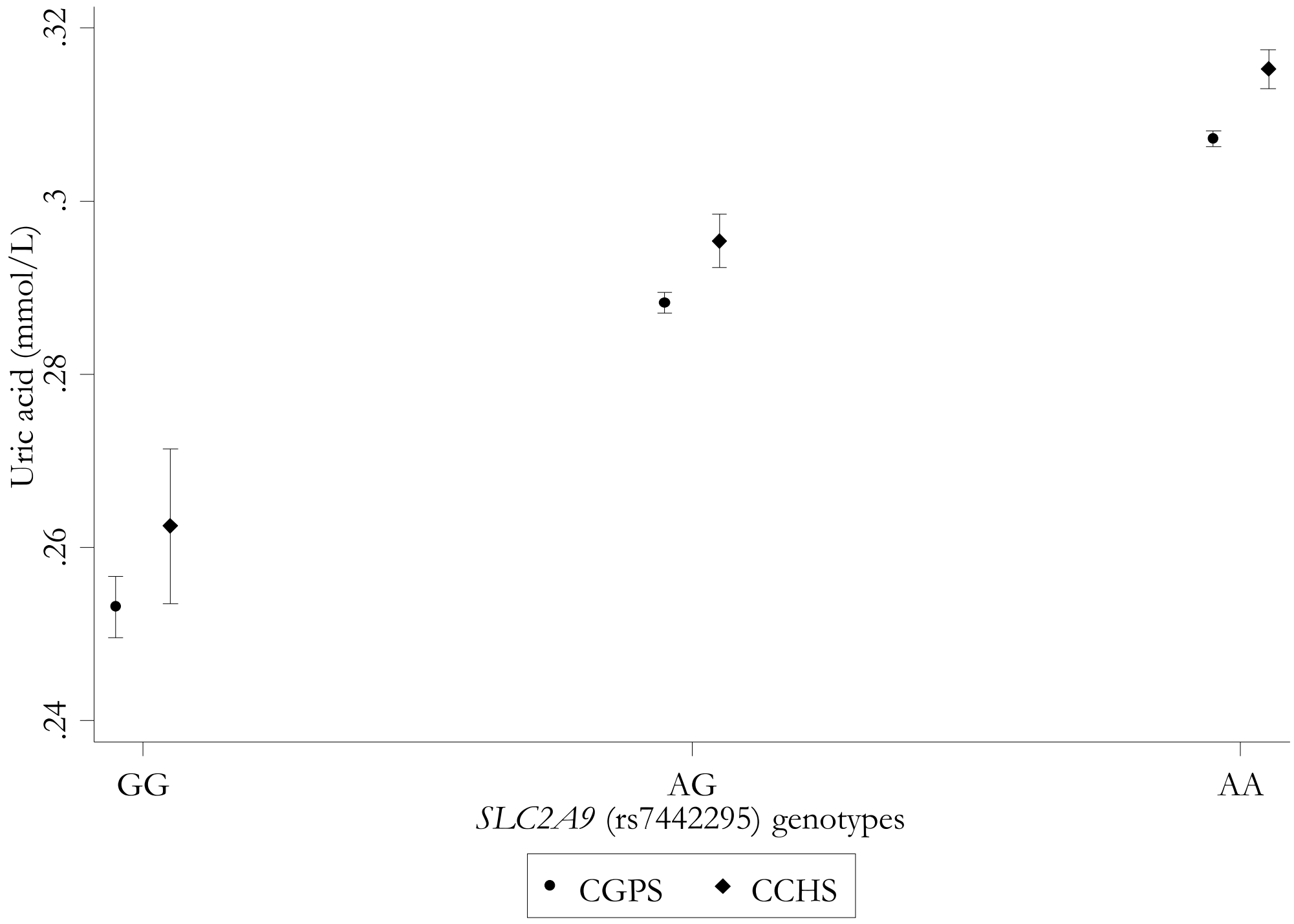

Supplementary Figure 5. Forest plots of observational and instrumental variable estimates of A) the effect of standardized urate and B) the effect of hyperuricemia status on body mass index (kg/m^2).

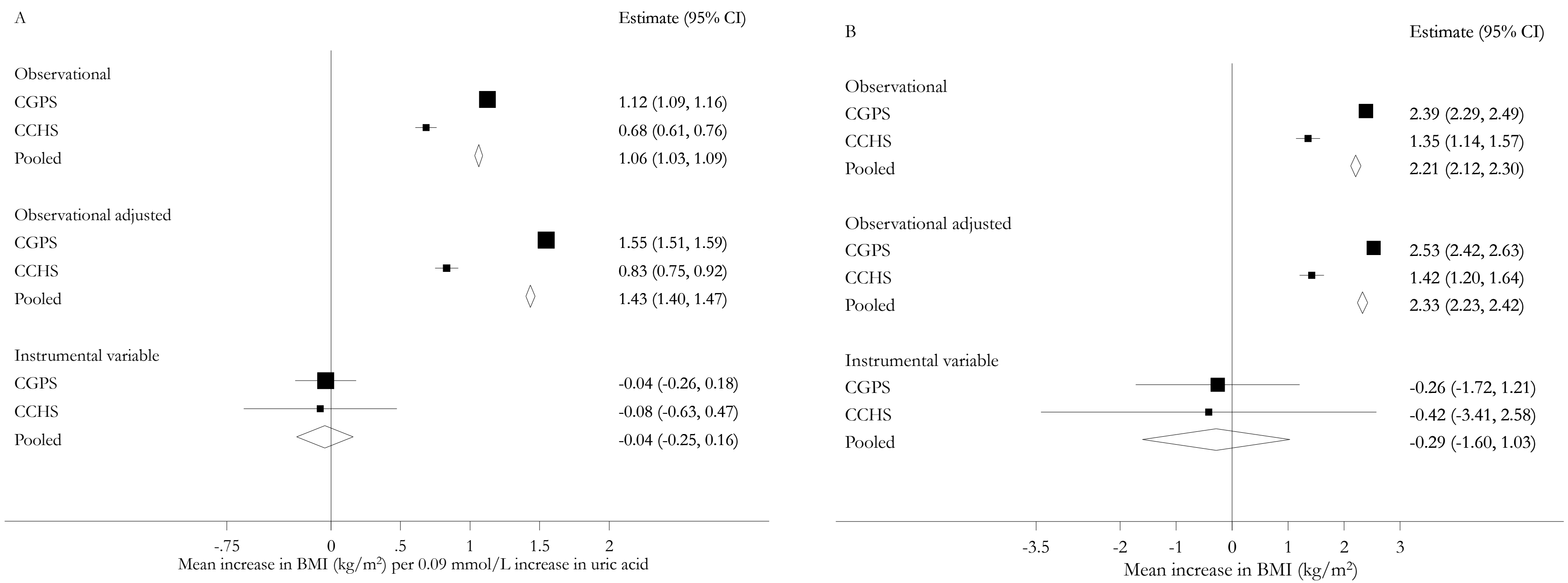

Observational adjusted estimates adjusted for age, sex, smoking, education, and income. CGPS: Copenhagen General Population Study; CCHS: Copenhagen City Heart Study; CI: confidence interval.
